# Disrupted architecture and fast evolution of the mitochondrial genome of *Argeia pugettensis* (Isopoda): implications for speciation and fitness

**DOI:** 10.1186/s12864-020-07021-y

**Published:** 2020-09-03

**Authors:** Jianmei An, Wanrui Zheng, Jielong Liang, Qianqian Xi, Ruru Chen, Junli Jia, Xia Lu, Ivan Jakovlić

**Affiliations:** 1 School of Life Science, Shanxi Normal University, Linfen, 041000 PR China; 2 Bio-Transduction Lab, Wuhan, 430075 Hubei PR China

**Keywords:** Barcode, Speciation, Decoupling of nuclear and mitochondrial evolution, Branch length, Crustaceans, Inversion of the origin of replication

## Abstract

**Background:**

*Argeia pugettensis* is an isopod species that parasitizes other crustaceans. Its huge native geographic range spans the Pacific from China to California, but molecular data are available only for a handful of specimens from North-American populations. We sequenced and characterised the complete mitogenome of a specimen collected in the Yellow Sea.

**Results:**

It exhibited a barcode (*cox1*) similarity level of only 87–89% with North-American populations, which is unusually low for conspecifics. Its mitogenome is among the largest in isopods (≈16.5 Kbp), mostly due to a large duplicated palindromic genomic segment (2 Kbp) comprising three genes. However, it lost a segment comprising three genes, *nad4L-trnP-nad6*, and many genes exhibited highly divergent sequences in comparison to isopod orthologues, including numerous mutations, deletions and insertions. Phylogenetic and selection analyses corroborated that this is one of the handful of most rapidly evolving available isopod mitogenomes, and that it evolves under highly relaxed selection constraints (as opposed to positive selection). However, its nuclear *18S* gene is highly conserved, which suggests that rapid evolution is limited to its mitochondrial genome. The *cox1* sequence analysis indicates that elevated mitogenomic evolutionary rates are not shared by North-American conspecifics, which suggests a breakdown of *cox1* barcoding in this species.

**Conclusions:**

A highly architecturally disrupted mitogenome and decoupling of mitochondrial and nuclear rates would normally be expected to have strong negative impacts on the fitness of the organism, so the existence of this lineage is a puzzling evolutionary question. Additional studies are needed to assess the phylogenetic breadth of this disrupted mitochondrial architecture and its impact on fitness.

## Background

Eukaryotes are invariably characterised by the co-existence of two different genomes within a single organism: nuclear and mitochondrial. The maintenance of evolutionary coadaptation between the two genomes, necessary for the interacting mitochondrial and nuclear components to reach their functional potential, called mitonuclear ecology, is an emerging topic in molecular biology [1–3]. There is accumulating evidence that mitochondrial DNA may have a disproportionately large role in generating Dobzhansky–Muller incompatibilities, and that mitonuclear compatibility may play a major role in speciation [4–9]. It has been proposed [4, 7] that this is the explanation for why *cox1* barcoding works so well in a majority of animal lineages, i.e. why conspecific sequences usually exhibit barcode similarity levels of 98% or more [10–12]. Intriguingly, for reasons that remain poorly understood, in some animal lineages barcoding seems to fail, which indicates that the threshold at which mitonuclear incompatibility produces speciation is often lineage-specific [4, 13, 14].

Mitochondrial genomes the crustacean order Isopoda exhibit architectural hypervariability and frequent inversions of the origin of mitochondrial replication (ORI) in comparison to most other crustacean lineages [15–23]. Three independent ORI events have been proposed so far in the evolutionary history of isopods [22]: 1) in the common ancestor of all isopods, resulting in inverted (positive) GC skews on the majority strand in the isopod clade in comparison to the ancestral crustacean skews (negative) [15, 24, 25]; 2) in the common ancestor of the entire Asellota (suborder) lineage, resulting in negative GC skews [15, 22]; and 3) in the common ancestor of families Cymothoidae and Corallanidae (suborder Cymothoida) [17, 21]. Apart from affecting the mitogenomic replication and (putatively) transcription regulation, ORI events can produce mutational bursts, which can hamper phylogenetic and other evolutionary analyses [22, 26]. Indeed, recently it was shown that these multiple ORI events produced strong homoplastic compositional biases in isopods, which in turn cause artefactual clustering in phylogenetic analyses [17, 21]. All these features make isopods an interesting model for studying the evolution of mitochondrial architecture [17].

Isopod species belonging to the superfamilies Bopyroidea and Cryptoniscoidea (suborder Cymothoida) have specialised for parasitizing (both as intermediate and definitive hosts) on other crustaceans. As they mostly parasitize on caridean shrimps, they are commonly referred to as epicarideans, but their exact taxonomic status (infraorder or suborder) remains debated [19, 27, 28]. *Argeia pugettensis* Dana, 1853 (Bopyroidea: Bopyridae: Argeiinae) is one of the two species currently recognised (Worms database) as valid within the genus (along with *A. atlantica*). It has a very wide host range, more than 20 species of crangonid shrimp species, and a massive circumboreal geographic range in the Pacific, from China to California [27, 29, 30]. Due to these factors and some morphological differences between northwestern (Asian) and northeastern (North-American) Pacific populations, it remains unclear whether these populations all belong to the same species, or whether the Asian populations may have to be renamed to *Argeia pingi* [30, 31]. Previously, taxonomic hypotheses for this genus were put forward exclusively on the basis of morphology, but this approach is often an unreliable taxonomic tool in parasitic crustaceans due to a limited number of taxonomically informative traits and host-induced morphological variability [19, 32, 33]. However, molecular data for this genus remain remarkably scarce: only two GenBank entries are currently (May 2020) available for the entire genus, *cox1* (mitochondrial) and *18S* (nuclear) genes (both for *A. pugettensis*), and 6 barcode (*cox1*) sequences in the BOLD database (*Argeia* spp.). Importantly, all of these sequences belong to populations sampled along the North-American continent, so the genetics of Asian populations remains completely unstudied. Furthermore, currently there is only one mitogenome available for the entire Bopyroidea superfamily, *Gyge ovalis* (Bopyridae) [19], so mitogenomic evolution also remains very poorly understood in this lineage. Finally, parasites are a good model for studying speciation processes because they have a high potential for diversification and specialization, including sympatric speciation [34, 35]. Therefore, as a parasitic species with a massive native range and notable host plasticity, *A. pugettensis* is also an interesting model for studying speciation. Herein, we sequenced and characterised the complete mitogenome of a (nominally) *A. pugettensis* specimen collected in the Yellow Sea in order to generate molecular data needed for pursuing two-pronged objectives of this study: 1) conduct molecular comparison of Asian and North-American populations of this nominal species, and 2) study the evolution of mitogenomic architecture in isopods.

## Results

### Low *cox1* barcode similarity with other conspecifics

**Fig. 1:**
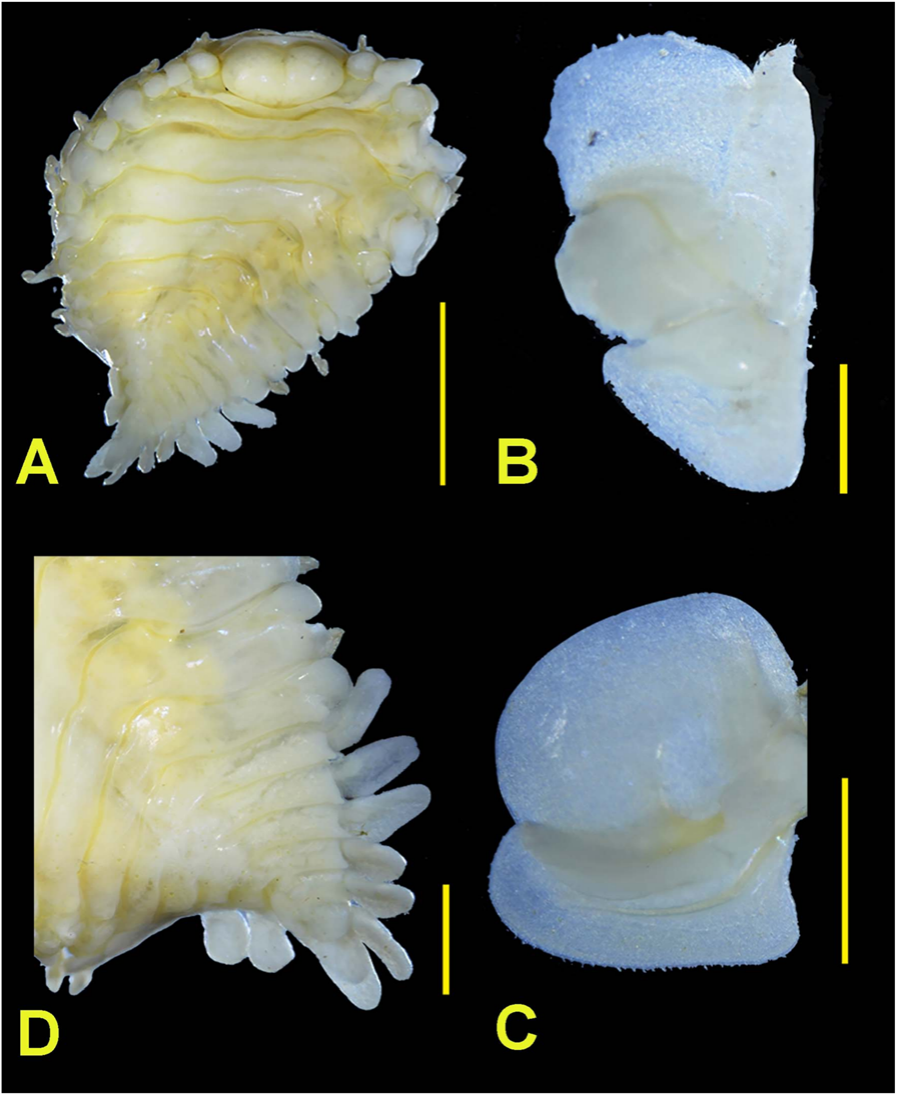
Photographs of the female specimen of *Argeia pugettensis* used for DNA extraction. **a** Dorsal view; **b** Maxilliped; **c** Internal view of left oostegite 1; **d** Dorsal view of pleon. Scale bars: A = 3 mm; B = 0.5 mm; C and D = 1 mm

**Fig. 2:**
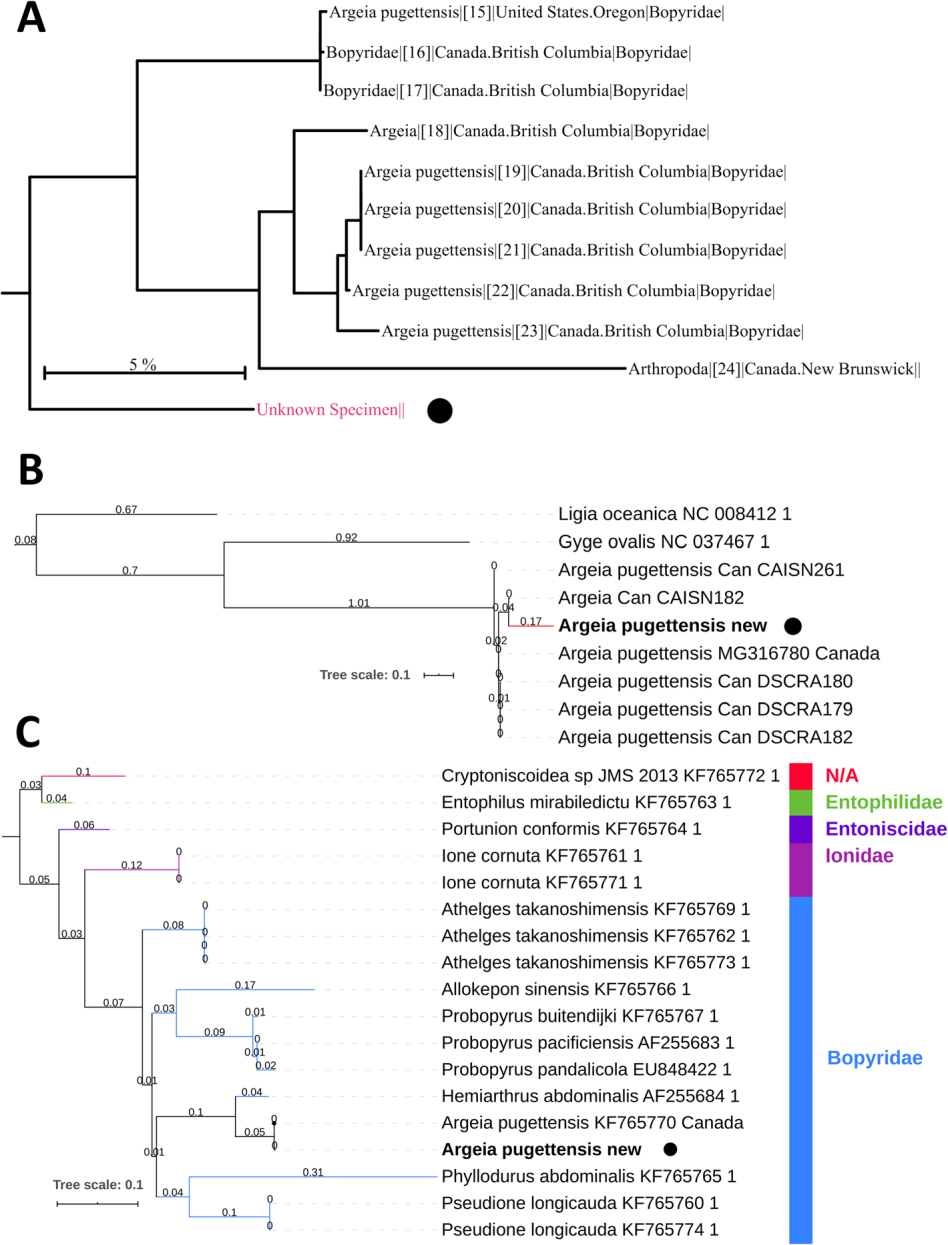
Barcoding results (**a**), and evolutionary rates of *18S* (**b**) and *cox1* (**c**) in *Argeia pugettensis* and other isopods. Barcoding was conducted using the BOLD database. The ML phylogenetic reconstructions were conducted using nucleotides of corresponding two genes. Lengths are shown on the branches, GenBank numbers next to species names, and isopod families to the right (**c**). Sequences belonging to the studied specimen are highlighted by a black dot (+ bolded)

The specimen (Fig. 1) was morphologically very similar to *A. pugettensis* described in [30], and *cox1* barcoding analysis in the BOLD database also identified *A. pugettensis* as the closest match, but with surprisingly low sequence similarity of only 87–89% (Fig. 2a). As conspecific barcode sequences usually exhibit similarity levels of 98% or more, this is unusually low for nominal conspecifics, but corresponds fairly well to inter-species congeneric similarities observed in other animal species [10–12]. Therefore, as all *Argeia* spp. sequences available in the BOLD database belong to specimens from the North-American populations (Fig. 2a), along with minor morphological differences between the two populations, barcoding analysis suggests that Asian populations should be elevated to a different species, putatively *A. pingi* [30, 31]. However, morphological differences are often not a reliable tool for distinguishing species of parasitic crustaceans due to host-induced morphological variability [33], and *cox1* barcode thresholds are often not reliable in lineages that exhibit elevated mitogenomic evolution rates [7, 36, 37]. Therefore, we set out to assess whether the *cox1* gene (and the entire mitogenome) of this species may be evolving at exceptionally elevated evolutionary rates, thus invalidating the applicability of standard barcode thresholds to this isopod lineage.

### Evolutionary rates

We conducted phylogenetic analyses using the (usually) most conserved mitochondrial gene *cox1* [38]. Dataset comprised of entire genes (extracted from the mitogenomic dataset) indicates that *cox1* of *A. pugettensis* is the fastest-evolving gene in the entire isopod dataset, closely followed by the only representative of the same family, *G. ovalis* (Additional file 1). To better assess the evolutionary rates within the family Bopyridae, we conducted the same analysis using barcode data (*cox1* fragment). This analysis showed that the studied sequence, i.e. the only representative of the Asian *A. pugettensis* populations, is evolving under elevated molecular rates in comparison to congenerics from North-American populations (Fig. 2b). The analysis nested the Chinese specimen within the *Argeia* clade, but this should be interpreted with some reservations due to limited phylogenetic signal contained within relatively short barcode sequences.

To assess whether the elevated *cox1* evolution rates are mirrored in the evolution of nuclear genes, we sequenced a fragment of the *18S* gene. The newly sequenced fragment was almost identical to the available sequence from a Canadian population (KF765770), with only two polymorphic nucleotides in the 805 bp-long fragment. Phylogenetic analyses confirmed that both available *A. pugettensis* (Canadian and Chinese) sequences evolve at almost identical rates (Fig. 2c). Moreover, their evolutionary rates were standard both within the epicaridean and isopod datasets (Fig. 2c, Additional file 2). Intriguingly, by far the longest branches in the isopod dataset were exhibited by Armadillidae, indicating exceptionally elevated evolutionary rates, followed by Cymothoidae and Limnoriidae. Among the Bopyridae, *Phyllodurus abdominalis* exhibited the longest branch (not available in the mitogenomic dataset).

**Fig. 3:**
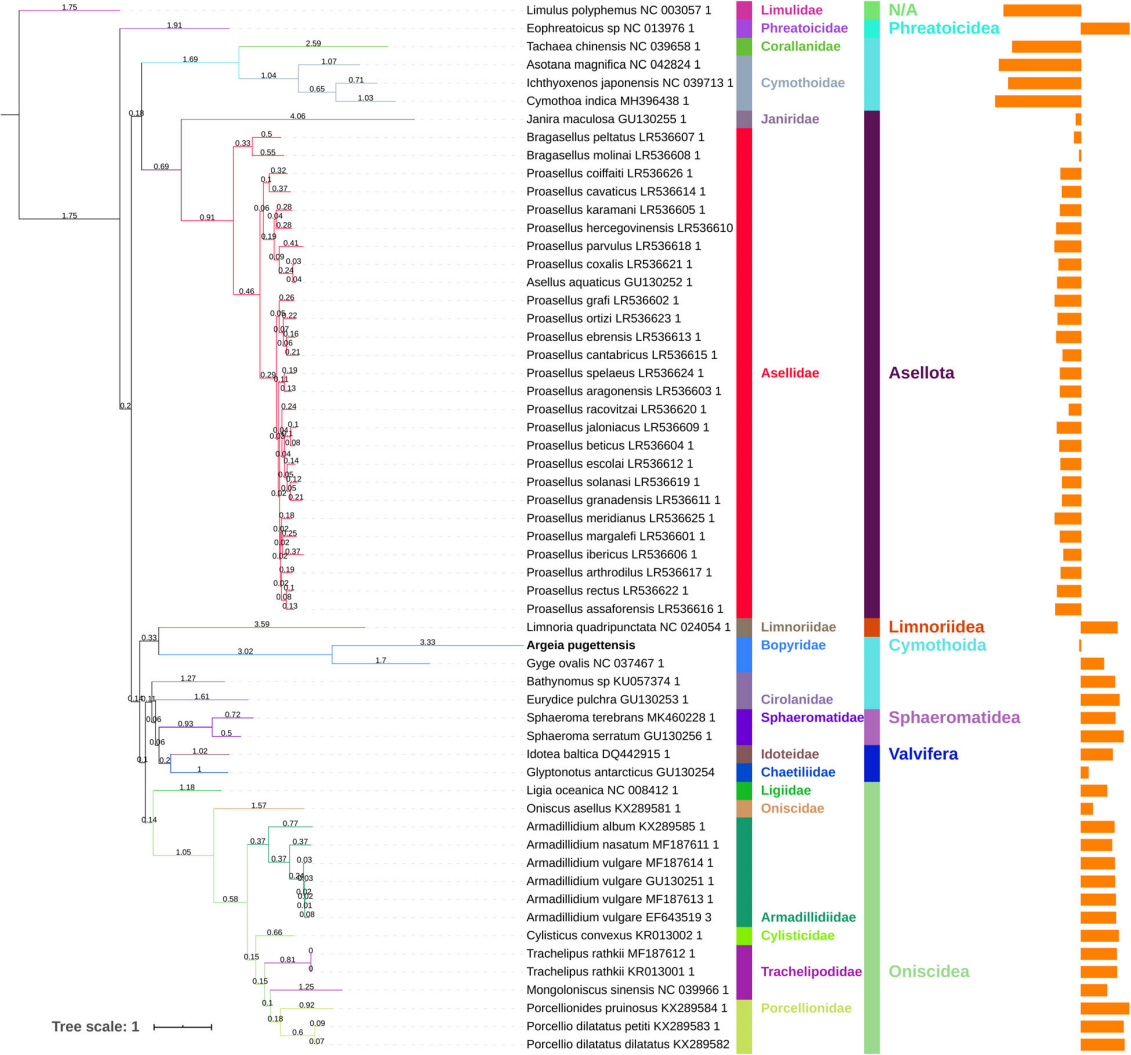
Branch lengths inferred using mitochondrial phylogenomics of Isopoda. Phylogenetic reconstruction was conducted using amino acids of concatenated 13 protein-coding genes and CAT-GTR algorithm implemented in PhyloBayes. The figure shows (from left to right): a phylogram with branch lengths shown, names of taxa with GenBank numbers for mitogenomes, Family, Suborder, and GC skew on the entire mitochondrial majority strand. *Limulus polyphemus* is the outgroup

Finally, to assess whether the elevated *cox1* evolutionary rates are mirrored in the entire mitogenome, we conducted phylogenetic analyses on all 13 PCGs, using both amino acids (Fig. 3) and nucleotides (Additional file 3). Both datasets produced the previously described base composition-driven artefact of Cymothoidae+Corallanidae clustering near the base of the isopod clade [17, 21], and revealed that elevated mitochondrial evolution rates are not limited to *cox1*: *A. pugettensis* had the longest branch, followed by *G. ovalis*. Elevated evolutionary rates were also exhibited by Limnoriidae (*Limnoria quadripunctata*), *Janira maculosa* (Asellota) and sister clades of Cymothoidae+Corallanidae. The two 13PCGs datasets (amino-acids and nucleotides) produced highly congruent results between each other, and in comparison to the *cox1* dataset. The only outlier was *J. maculosa*, whose elevated mitogenomic evolution was not mirrored in the *cox1* dataset. As this mitogenome is incomplete (only about 9 Kbp), we suspect that this may be an artefact. In partial agreement with previous findings [39], the slowest evolutionary rates in the 13PCGs dataset were observed in: *Ligia oceanica* (Oniscidea), *Bathynomus* sp. (Cymothoida), and Valvifera. In comparison, in the *cox1* dataset, the slowest rates were exhibited by *Bragasellus* sp. (Asellota) and *Ligia oceanica*.

### Selection force analyses: relaxed vs. positive selection

We hypothesised that the reason for the highly divergent *cox1* and overall mitogenomic sequence of *A. pugettensis* lies in the relaxed negative (purifying) selection constraints, as opposed to positive (Darwinian) selection. To test this hypothesis first we used RELAX algorithm in the exploratory mode (all isopod mitogenomes selected as test branches) on the *cox1* dataset. This tool calculates k value, where k > 1 indicates intensified selection and k < 1 indicates relaxed along the test branches [40]. This analysis identified *L. quadripunctata*, *Tachaea chinensis* (Corallanidae), *J. maculosa* and *A. pugettensis* as species with lowest k values (0.20–0.22). Only five other species had k values < 1, whereas among the remaining species 19 had 1 < k < 5, and 27 had k > 5 (Additional file 4). In the 13 PCG dataset, the above four species were among the 12 species in the narrow k range of 0.19–0.23, but a majority of species (32) was in the k > 5 range. These results correspond relatively well to the branch lengths, but statistical test for relaxation was non-significant with *A. pugettensis* selected as the test branch, probably as a result of a handful of other species exhibiting similar k values. We further analysed the *cox1* dataset using BUSTED, which provides a gene-wide test for positive selection [41], to corroborate that elevated *cox1* evolution rates in *A. pugettensis* are attributable to relaxed selection pressures, and not to positive selection. With *A. pugettensis* selected as the test branch, BUSTED found no evidence (*p*-value = 0.50) that any sites have experienced positive selection.

We also applied the free-ratio branch evolutionary model [42] implemented in *ete-evol* to infer the numbers and ratios (ω) of synonymous (dS) and nonsynonymous (dN) mutations in the *cox1* dataset: *L. quadripunctata* (dN = 12.89 / dS = 199.46, ω = 0.065), *J. maculosa* (8.16/168.4, ω = 0.049), and *A. pugettensis* 8.73/156.6, ω = 0.056) had the highest numbers of mutations, with relatively similar ω values (Additional file 5). Intriguingly, *Glyptonotus antarcticus* exhibited a larger number of dN, but much lower number of dS (9.06/126.66), and correspondingly somewhat higher ω values (0.072), than *A. pugettensis*, which is indicative of positive selection. As this species is an extremophile (seas around the Antarctica), this raises a possibility that these may be signatures of adaptive evolution to the extreme habitat. Although these analyses indicate that *A. pugettensis* is merely among a handful of fastest-evolving isopods with respect to its mitochondrial genome, they depend on codon alignments, so they fail to measure the evolutionary signal in such features as highly truncated genes in this species.

### Mitogenomic architecture: missing genes

**Fig. 4:**
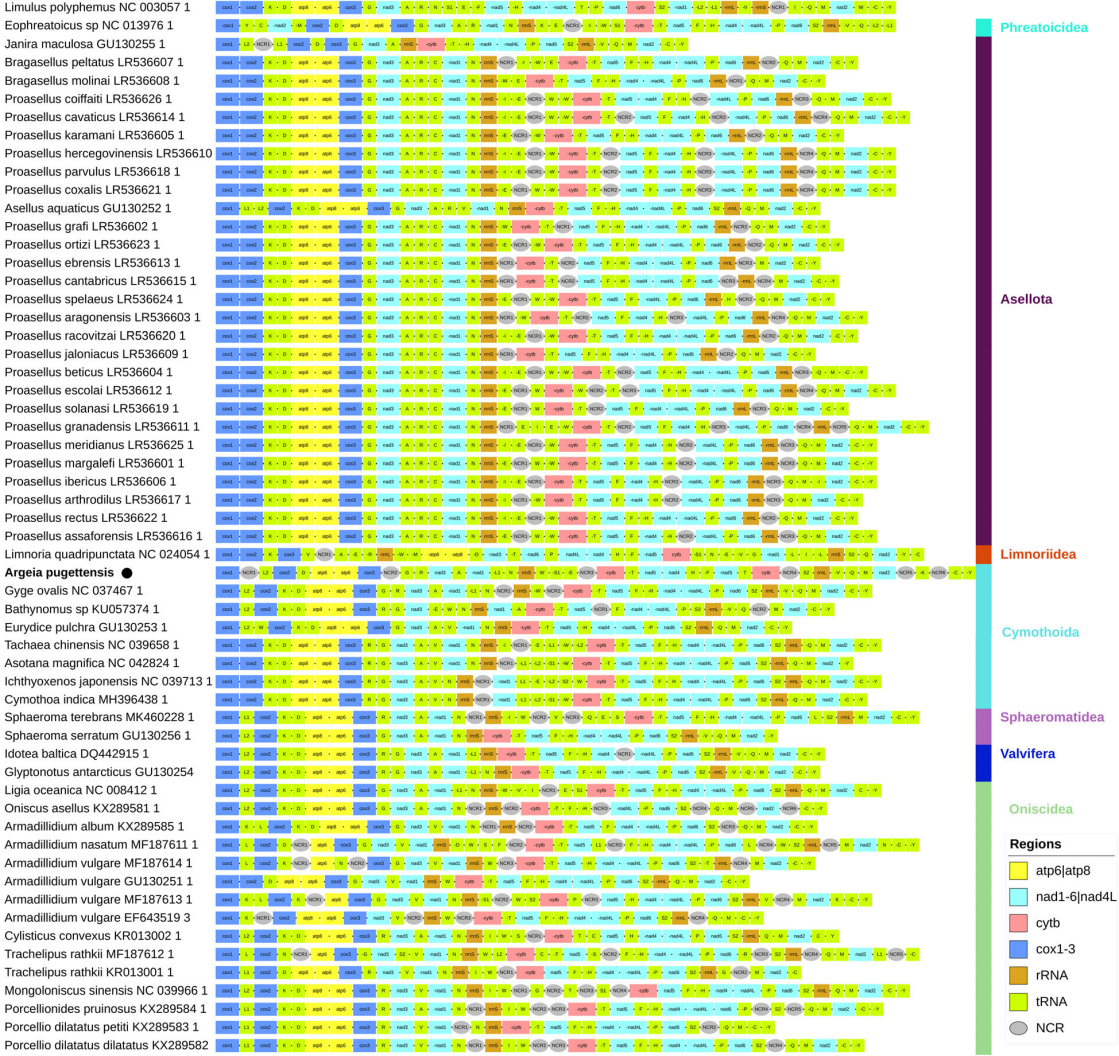
Gene orders in the mitogenomes of Isopoda. A dash (−) before the gene name indicates that the gene is encoded on the minus strand. NCR indicates a non-coding region > 100 bp. *Argeia pugettensis* is bolded and marked with a black dot

**Fig. 5:**
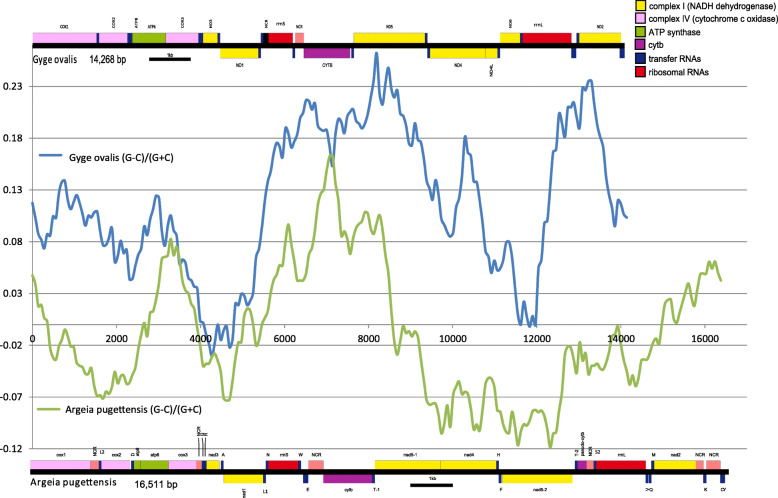
Architecture and cumulative GS skew plots on the entire plus strand of mitogenomes of *A. pugettensis* and *Gyge ovalis*. Window size was manually set to 1500 bases, and step size automatically to 79 bases. Genes shown above the black line are encoded on the plus strand, and those on the minus strand below. Gene legend is shown in the figure. NCR indicates a non-coding region larger than 100 bp

We conducted a number of comparative mitogenomic analyses to assess whether the elevated sequence evolution rates of the mitogenome of *A. pugettensis* are reflected in the evolution of its architecture. We identified 35 genes in the mitogenome, but only 33 of those were unique (two duplicated genes), so four genes are missing from the standard mitogenomic set [43]: *trnP, trnI, nad4L and nad6* (Figs. 4 and 5; Additional file 6: Table S2). Even if we disregard the duplicated and missing genes, the mitogenome exhibited a unique gene order in the available isopod mitogenomic dataset, with a large number of tRNA gene rearrangements in comparison to *G. ovalis* (Fig. 4). The exceptionally high gene order rearrangement rate in isopods is particularly well exemplified by the hypervariability exhibited by 24 congeneric *Proasellus* spp. mitogenomes.

*trnI*, *trnW* and *trnE* are commonly missing from isopod mitogenomes [16, 19], but protein coding genes seem to be missing from only a handful of other isopod species: *nad2* in *Proasellus beticus*, *nad4* in *Proasellus spelaeus*, and *atp8* from several *Armadillidium* species (Fig. 4; Additional file 7). Unfortunately, as these remain either unpublished, or authors did not discuss this further [44, 45], we cannot assess with certainty whether these might be sequencing/annotation artefacts. As the *nad4L-trnP-nad6* segment is conserved in most isopods (Fig. 4), we suspected that the mitogenome might be fragmented into two chromosomes. To check this, we designed a set of degenerate primers to match *nad4L* and *nad6* genes (two primers for each gene) in other isopods (Additional file 6: Table S1), but this did not produce any observable PCR products. Further, we checked whether the missing genes may have migrated to the nuclear genome [46], for which we conducted PCR walking experiments using the forward primers from the previous step (Additional file 6: Table S1). Finally, we amplified the entire mitogenome in two steps to confirm that the sizes of amplicons correspond to the expected total mitogenome size by PCR (Additional file 6: Table S1 and Figure S1). As we made sure that all amplified segments of the mitogenome overlap by about 100 bp, and that overlaps match, we can claim with some confidence that the sequenced and assembled mitogenome is complete and circular. We also assessed the possibility of heteroplasmy: the existence of different mitochondrial genomes within the same organism. When two types of mitochondrial architectures exist within a single organism, it sometimes shows as a double PCR band. If this organism possessed both normal and disrupted mitochondrial genomes, their sizes and sequences would be different. This in turn would produce double bands in some PCR reactions and noisy sequencing electropherograms (double peaks), but our amplification and sequencing experiments did not produce any indications of heteroplasmy. This was further corroborated by the two-step re-amplification of the mitogenome described above. We also checked whether the missing genes may be in one of the six large (>NCRs) in the mitogenome by attempting to align them with *nad6* and *nad4L* genes from other isopods using the ‘-adjustdirectionaccurately’ function in MAFFT and using algorithms available in Geneious, but none of the NCRs exhibited any notable similarity to the two genes. Finally, we inspected these NCRs for open reading frames (ORFs). Using relaxed settings, we identified 12 putative ORFs ranging from 50 to 96 AAs. As we could not identify any ORFs > 50 AAs in the NCR2, we lowered the size threshold and identified a putative 43 AA ORF. We attempted to align all 13 ORFs with the entire isopod datasets for these two missing genes. *nad4L* alignment did not produce any notable similarity, but *nad6* alignment revealed what might be a highly degraded fragment of the 3′-end of *nad6*, comprising some 15–20 amino acid residues (Additional file 8). Intriguingly, it was found in the 51 aa ORF that comprised most of the NCR between pseudo-*cytb-2* and *trnS2*, the location that perfectly corresponds to the position of *nad6* in many isopod mitogenomes, including that of *G. ovalis* (Fig. 4).

### Mitogenomic architecture: a large duplicated segment and divergent genes

A segment comprising cytb^(−)^-trnT-1^(−)^-nad5–1^(+)^ was duplicated, translocated and underwent a double-stranded inversion: nad5–2^(−)^-trnT-2^(+)^-pseudo-cytb^(+)^ (Figs. 4 and 5, Additional file 6: Table S2). This gene cartridge is highly conserved in isopods, so we can infer the evolutionary history (original and copy) of the two segments with confidence. A major part of the *cytb* copy was lost during this rearrangement; we identified only a perfectly conserved 212 bp-long 5′-end segment. The 168 bp-long NCR between the *cytb* fragment and *tRNA-Ser (tga)* contained a few motifs that partially corresponded to the complete *cytb*, but with large deletions between. The central segment of both copies of *nad5* was highly conserved, with only four base mutations, but the copy (*nad5–2*) was truncated about 20 AAs at the 5′ end, whereas the original gene (*nad5–1*) had 3′ end truncated by about 55 AAs. Alignment with orthologues revealed that similar 5′ truncations are relatively common, whereas none exhibit a similar 3′ truncation, from which we infer that the copy of this gene, *nad5–2*, is the functional paralogue.

Three genes were truncated by introduction of the TAG stop codon at the 3′ end in comparison to many other isopod orthologues: *nad3, cox1* and *cox3*. *Nad3* was truncated by about 40–50 bases, *cox1* by about 110, whereas the entire 3′ half of *cox3* was highly divergent from other orthologues (5′ half was highly conserved), and the gene was ≈130 bases shorter than orthologues from the same suborder (Additional file 7). This is highly unusual, especially for *cox1*, which is usually among the most highly conserved mitochondrial genes [38], but there are no indications of the stop codon being a sequencing artefact (Additional file 6: Figure S2). As codon reassignment and suppression occur comparatively often in mitochondrial genomes [47, 48], we considered a possibility of TAG stop codon suppression. We identified a TAA codon within the NCR downstream from *cox1*, the use of which would extend the gene by 120 bp, but these extra 40 AAs did not exhibit any similarity with orthologues (Additional file 6: Figure S3), from which we inferred that truncated gene is a more parsimonious option. Apart from the large duplicated palindromic segment discussed above, we found only one additional palindromic repeat > 15 bp (31 bp) (Additional file 6: Table S3).

### Base composition skews and ORI events in isopods

The common ancestor of all isopods is believed to have undergone an ORI, resulting in inverted (positive) skews in the isopod clade in comparison to the ancestral crustacean skews (negative). This was subsequently followed by two independent ORI events, both producing negative overall skews: one in the Asellota lineage, and the other in the common ancestor of families Cymothoidae and Corallanidae (Cymothoida) [15, 21, 22]. The overall skew of the new mitogenome is negative, but very close to zero (− 0.008), which is comparable to some Asellota species, but very different from the closest relative, *Gyge ovalis* (0.118) (Figs. 4 and 5). This indicates that *A. pugettensis* exhibits the fourth proposed ORI event in the evolutionary history of isopods. Although most ORI events in crustaceans can be associated with inversions of *rrnS*, adjacent to the control region in the ancestral crustacean gene order [49], isopods are a partial exception from this: only the initial ORI event in the ancestral isopod was accompanied by a strand switch of *rrnS* (minus to plus), but subsequent two ORI events were not accompanied by strand switches of this gene (it remains on plus strand in all isopods) [22]. To explain this, ORI events comprising only the CR were proposed for the two lineages with inverted skews [22]. Apart from the strand switch of the duplicated fragment, the mitogenome of *A. pugettensis* also does not exhibit synapomorphic strand switches of genes that would be indicative of an ORI. To get a better resolution, we plotted cumulative skews against the mitogenomic map, and compared them to those of *G. ovalis* (Fig. 5). Both mitogenomes exhibited noisy plots, with skews of *A. pugettensis* frequently switching between positive and negative values. In the absence of numerous recent strand switches, this can be explained either by a highly disrupted replication mechanism, with the mitogenome being replicated in several fragments, or by an evolutionary recent ORI event, resulting in only partially inverted skews [22]. The latter option is the most likely explanation for its skew pattern, as we can infer with some confidence that this putative ORI is specific to this genus (or subfamily), and therefore it should be the most recent in evolutionary terms among the four ORIs in isopods. Sequencing of further mitogenomes from this isopod (sub) family is needed to corroborate this putative ORI and infer its exact evolutionary timing.

## Discussion

### Disrupted mitogenomic architecture and expected impacts on organism’s fitness

Mitogenomic architecture rearrangements are nonadaptive events expected to have a negative effect on organism’s fitness due to their impacts on the genome replication and transcription mechanisms, as well as disruptions in the gene expression co-regulation both within the mitochondrial genome and between the two genomes (mitonuclear ecology); as a result, mitogenomes generally have highly conserved architecture [2, 50, 51]. However, it appears that many metazoan lineages, such as isopods, manage to thrive regardless of the high frequency of mitogenomic architectural rearrangements. Even among the isopods, the architecture and sequence of the mitogenome of this (nominally) *A. pugettensis* specimen collected in the Yellow Sea evolve under exceptionally relaxed purifying selection pressures. Many genes had highly divergent sequences in comparison to closely related isopod orthologues, including numerous mutations, deletions, insertions, and even major sequence truncations. It also exhibited multiple gene order rearrangements, including a putative control region inversion, resulting in an inverted (negative) overall GC skew on the major strand in comparison to most other isopods. Furthermore, it exhibited a large (2 K bp) palindromic duplication comprising three (incomplete) genes, so it possesses one of the two largest mitogenomes in the available isopod dataset, along *L. quadripunctata* (16,511 and 16,515 bp respectively). Although we cannot completely exclude a possibility that fast-evolving mitochondria may confer some advantages with regard to the host range and host-parasite arms race, the most parsimonious explanation for these unusual features is nonadaptive, i.e. exceptionally relaxed selection pressure on the mitochondrial genome [52, 53].

While gene order rearrangements and multiple large NCRs are common in isopods, duplications of genes are rare and limited to a handful of tRNA genes (Fig. 4). Although such large duplicated genomic segments comprising PCGs have been observed in isolated lineages [54–58], they are generally very rare in metazoans [59]. Regardless of their rarity, such duplications are not expected to produce debilitating effects on the functioning of mitochondria - whereas missing PCGs are. As a result, *atp6* and *atp8* aside [43, 59, 60], PCGs are almost never missing from metazoan mitochondrial genomes. Along with the missing genes, highly divergent and truncated sequences of PCGs of the mitogenome of *A. pugettensis* would be expected to further reduce the efficiency of ATP production [61–63]. Therefore, the disrupted architecture of this mitogenome would be expected to negatively the fitness [61, 64] of *A. pugettensis* populations that possess it. Alternatively, we also have to consider a possibility that reduced metabolic efficiency of this disrupted mitogenome has negligible impacts on the fitness of this species. As reduction of numerous biological processes is a hallmark of the parasitic lifestyle [65], a possible explanation would be that metabolic efficiency has limited impacts on fitness in parasitic species. There is some support for this hypothesis from previous studies, some of which found elevated evolutionary rates in mitochondria of some parasitic lineages [26, 66–68]. However, this is not a universal rule, as in some lineages parasitism seems to have negligible impacts on the evolution of mitochondrial DNA [66, 67]. From either perspective, the existence of this lineage is an interesting evolutionary phenomenon.

Deletions comprising segments or entire mitochondrial genes have been observed in some laboratory strains of the nematode *Caenorhabditis* sp. [61, 69]. Despite the fact that such deletions strongly affect the fitness, they sometimes reach high intracellular frequency and persist over long periods in populations [61]. However, aside from these being laboratory circumstances, these cases were all characterised by heteroplasmy, wherein both normal and mutant-type mitochondria exist within the same cell. We did not find indications of heteroplasmy during the amplification and sequencing of this mitogenome. While we cannot fully exclude a possibility that non-disrupted mitochondrial genomes exist in very small numbers, sufficiently small to not show in our amplification and sequencing experiments, such tiny proportions of mitochondria would not have major impacts on the metabolic efficiency of this organism.

We cannot exclude a possibility that, as a result of parasitic life style of this species, the disrupted architecture may have minimal impacts on its fitness. However, a similar phenomenon may have been observed in a free-living crustacean species, European lobster *Homarus gammarus* (Nephropidae). The first sequenced mitogenome of *H. gammarus* was missing the *nad2* gene [70], but a subsequent study identified all standard mitochondrial genes and very different architecture in the mitogenome belonging to a different specimen [71]. Additionally, the two mitogenomes exhibited different GC skews (− and +) [22]. Zhang et al. proposed that, assuming that this was not a sequencing and assembly artefact, this suggests that some lineages (populations or merely specimens) within a species may undergo major mitogenomic architecture disruptions, but continue to be viable for certain periods of time despite the presumably strongly reduced fitness caused by the loss of PCGs [22]. In this free living species, metabolic efficiency would be expected to strongly impact the fitness. However, we should note that lobsters are known for their relative longevity [72]; and as mitochondrial ROS production is associated with ageing [63], we cannot reject a possibility that reduced metabolic activity may confer some other selective advantages to this species.

As our barcode sequence analyses indicate that the mitogenome of this (first Asian) *A. pugettensis* lineage evolves at a higher rate than the North-American populations, we hypothesise that the latter do not share the highly disrupted mitogenomic architecture. To test this hypothesis it is necessary to assess the phylogenetic breadth of this disrupted mitogenomic phenotype. Currently we do not know whether it may be present in a single specimen, a single lineage, population, or in most of the Asian (western Pacific) populations.

While the low similarity of *cox1* barcode sequences between nominally conspecific Asian and North-American populations of *A. pugettensis* appears to suggest that the former should be assigned to a different species, almost identical sequences of the nuclear *18S* gene indicate that this may be merely an artefact caused by elevated mitochondrial mutational rates. Therefore, our analyses indicate that the mitogenome of Asian *A. pugettensis* populations may be evolving under highly relaxed purifying selection pressures in comparison to other isopods and North-American conspecifics, but this does not appear to be reflected in the mutation rates of nuclear genome. Such decoupling of mutational rates between mitochondrial and nuclear genomes was recently reported in another isopod suborder, Asellota [73]. Herein, we also observed it for Armadillidae, whose mitogenomes are evolving under strong purifying selection pressures (slowly), but their *18S* gene appears to be evolving at elevated rates. More nuclear genes are needed to confirm this. The decoupling of evolutionary rates does not seem to be the rule in isopods, as our findings support a recent observation that both mitochondrial and nuclear genes of Cymothoidae and Limnoriidae evolve at elevated rates [21]. Elevated evolutionary rates of Bopyridae, Limnoriidae, and to an extent Cymothoidae+Corallanidae, may partially explain their exceptional phylogenetic instability [21, 74]. The decoupling of rates in this *A. pugettensis* lineage is most likely caused by relaxed purifying selection pressures on its mitogenome. This is in agreement with the observation that *cox1* barcoding often seems to fail for sedentary animal lineages [7], so we hypothesise that a reduced selection for locomotory capacity in this parasitic species may have decreased the strength of selection on mitonuclear coadaptation.

Unfortunately, our findings are severely hampered by the limited amount of data (a single specimen) collected for this study, so the study opens more questions than it manages to resolve. The questions that remain open due to these limitations are: 1. The taxonomic status of Asian populations and the extent of gene flow between different populations; 2. Geographic range of the disrupted mitochondrial architecture; 3. The functionality of the disrupted mitochondrial genome identified in this study. Therefore, these questions should be studied using representative samples spanning the entire geographic range of this nominal species. Ideally, they should combine both mitochondrial and nuclear molecular markers, and use them to test the magnitude of gene flow between these populations.

## Conclusions

Mitochondrial functioning depends on the interaction between nuclear and mitochondrial genomes, and there is increasing evidence that their incompatibilities may have disproportionately large role in speciation. Here we report a mitochondrial genome of a parasitic isopod crustacean, that evolves under exceptionally highly relaxed selection pressure, which resulted in highly divergent gene sequences, a large genomic duplication, and missing protein coding genes. Our analyses indicate that this is not reflected in the evolution of it nuclear genome, and that it is limited to Asian lineages of this species. As such highly disrupted mitogenomic architecture would be expected to affect the efficiency of ATP production, and thus affect the fitness of the organism, the existence of this lineage is a puzzling evolutionary question.

## Methods

### Sample, sequencing, assembly and annotation

A single *A. pugettensis* specimen was procured from a crangonid crustacean host *Crangon hakodatei* Rathbun 1902 caught in the Yellow Sea (27°20′N, 123°30′E). It was identified morphologically following the descriptions in [30, 31, 75, 76], and via *cox1* barcoding using the BOLD database [77]. As the animal handling included only unprotected invertebrates (crustaceans), no special permits were required to retrieve and process the sample.

DNA extraction, amplification, sequencing and mitogenome assembly were conducted closely following the methodology described before [16, 17]: DNA was extracted from the complete specimen using AidLab DNA extraction kit (AidLab Biotechnologies, Beijing, China), and mitogenome amplified and sequenced using 14 primer pairs (Additional file 6: Table S1). We designed the primers to produce amplicons that overlap by approximately 100 bp. PCR reaction mixture of 50 μL comprised 5 U/μL of TaKaRa LA Taq polymerase (TaKaRa, Japan), 10 × LATaq Buffer II, 2.5 μM of dNTP mixture, 0.2–1.0 μM of each primer, and 60 ng of DNA template. PCR conditions were: denaturation at 98 °C for 2 min, followed by 40 cycles of 98 °C for 10 s, 50 °C for 15 s, and 68 °C for 1 min/kb. PCR products were sequenced using the same set of primers and Sanger method. A fragment (805 bp) of the nuclear *18S* gene was amplified and sequenced using a similar methodology (primers in the Additional file 6: Table S1). Sequences were quality-proofed via visual inspection of electropherograms, and their identity confirmed using BLAST [78]. During the assembly of the mitogenome, conducted using DNAstar v7.1 [79], we made sure that overlaps were identical, the mitogenome circular, and that no *numt*s were incorporated. Protein-coding genes were approximately located using DNAstar and then manually fine-tuned according to the orthologous sequences using BLAST and BLASTx. The missing PCGs were additionally searched using algorithms implemented in Geneious 8.15 (https://www.geneious.com). tRNAs were identified using tRNAscan [80] and ARWEN [81] tools, and the two ribosomal RNAs were precisely manually annotated via a comparison with orthologues. The mitogenome of *Gyge ovalis* [19], the only available Bopyroidea representative, was used as the template for assembly and annotation. The annotation recorded in a Word (Microsoft Office) document was parsed and extracted using PhyloSuite [82]. The same program was used to generate the file for submission to GenBank.

### Comparative mitogenomic, phylogenetic, sequence and selection analyses

PhyloSuite was also used to retrieve all available isopod mitogenomes from the GenBank (Additional file 7), semi-automatically re-annotate ambiguously annotated tRNA genes with the help of the ARWEN output, standardise the annotation (gene names), extract mitogenomic features and generate comparative tables, calculate skews, translate genes into amino acid sequences, concatenate alignments and prepare input files for its plug-in programs: MAFFT [83], used to align sequences, and IQ-TREE [84], used to conduct orientational phylogenetic analyses applying the Maximum Likelihood (ML) algorithm. For subsequent phylogenetic analyses, we used amino acid sequences of concatenated 13 protein-coding genes (PCGs) in combination with the CAT-GTR model in PhyloBayes-MPI 1.7a, designed to account for compositional heterogeneity [85], as a study has shown that this was the most successful strategy for alleviating the compositional bias-driven long-branch attraction artefacts in isopod mitochondrial phylogenomics [21]. The PhyloBayes analysis was conducted on the Cipres server [86], with default parameters (burnin = 500, invariable sites automatically removed from the alignment, two MCMC chains), and automatically stopped when the conditions considered to indicate a good run were reached: maxdiff < 0.1 and minimum effective size > 300 (PhyloBayes manual). Sample size was 17,889 when the terminator got a signal. Phylogenetic analyses of *cox1* and *18S* datasets were conducted partly as described above with the following differences: Flowchart mode was used in PhyloSuite; these alignments were very gappy, so they were trimmed using trimAI [87]; the optimal model was selected using ModelFinder [88]; and phylogeny was inferred using IQ-TREE with 10^5^ Ultrafast bootstraps [89]. For *cox1* dataset, we added six additional *Argeia* spp. (Additional file 7) sequences available in the BOLD/GenBank databases. For the *18S* dataset, we retrieved the dataset designed to correspond as closely as possible to the available mitogenomic dataset in a previous study [21], and added all available Epicaridea sequences, including a sequence from an *A. pugettensis* specimen collected in Canada (Additional file 7). Phylograms and gene orders were visualized and annotated (using files generated by PhyloSuite) in iTOL [90]. Detailed mitochondrial architecture was visualised using OGDRAW [91], and cumulative skews plotted using DAMBE7 [92]. Palindromes were searched using Palindrome Analyzer [93], and ORFs using NCBI’s ORF Finder. Selection pressures were studied using *ete-evol* tool in ETE 3 [94], and two tools available from the Datamonkey server [95]: RELAX [40] and BUSTED [41].

## Supplementary information

**Additional file 1 ***cox1*-based phylogenetic analysis of the entire isopod dataset. The analysis was conducted using Maximum Likelihood algorithm and nucleotide sequences. Lengths are shown on the branches, GenBank numbers next to species names, and taxonomic identity (family and suborder) to the right. The studied specimen is highlighted by a black dot (+ bolded).

**Additional file 2 ***18S*-based phylogenetic analysis of the entire isopod dataset. The analysis was conducted using Maximum Likelihood algorithm and nucleotide sequences. Lengths are shown on the branches, GenBank numbers next to species names, and taxonomic identity (family and suborder) to the right. The studied specimen is named “*Argeia pugettensis* 18S new”.

**Additional file 3 **Branch lengths inferred using mitochondrial phylogenomics of Isopoda (nucleotides dataset). Phylogenetic reconstruction was conducted using nucleotides of concatenated 13 protein-coding genes and ML algorithm. The figure shows (from left to right): a phylogram with branch lengths shown, names of taxa with GenBank numbers for mitogenomes, GC skew on the entire mitochondrial majority strand, and suborder. *Limulus polyphemus* is the outgroup.

**Additional file 4 **Detailed results of the RELAX analysis of *cox1* dataset.

**Additional file 5 **Nonsynonymous and synonymous mutations in the *cox1* gene of isopod mitogenomes. The numbers of nonsynonymous (dN) and synonymous (dS) mutations are shown in grey above the branches (dN/dS), and their ratios (ω) in red, below the branch. The numbers were inferred using free-ratio branch evolutionary model implemented in the *ete-evol* tool. The phylogram shown in Fig. 3 was used for the analysis. Corresponding GenBank numbers are shown next to species names.

**Additional file 6 **Primers, organization, size confirmation, *cox1* sequencing chromatogram and palindromic repeat analysis.

**Additional file 7.** Mitogenomic dataset, comparative mitogenomics tables, and alignments used for phylogenetic analyses.

**Additional file 8 **Alignment of the 3′ end of selected isopod *nad6* orthologues with the *A. pugettensis* ORF identified in the NCR between pseudo-cytb-2 and trnS2.

## Data Availability

All data generated or analysed during this study are included in this published article, its supplementary information files and the NCBI’s GenBank repository under the accession numbers MG753775 (the complete mitogenome) and MT450212 (*18S*). GenBank accession numbers of all sequences used in the analyses (complete mitogenomes, *cox1* and *18S* sequences) are available in the Additional file 7.

## Abbreviations

AA: Amino acid
NCR: Non-coding region
ORF: Open-reading frame
ORI: Inversion of the origin of replication
PCG: Protein-coding gene
