# Supplementary figures and images for: Disrupted architecture and fast evolution of the mitochondrial genome of Argeia pugettensis (Isopoda): implications for speciation and fitness
Source: BMC Genomics. 2020 Sep 3;21:607. doi: 10.1186/s12864-020-07021-y (PMC7469299; doi:10.1186/s12864-020-07021-y)

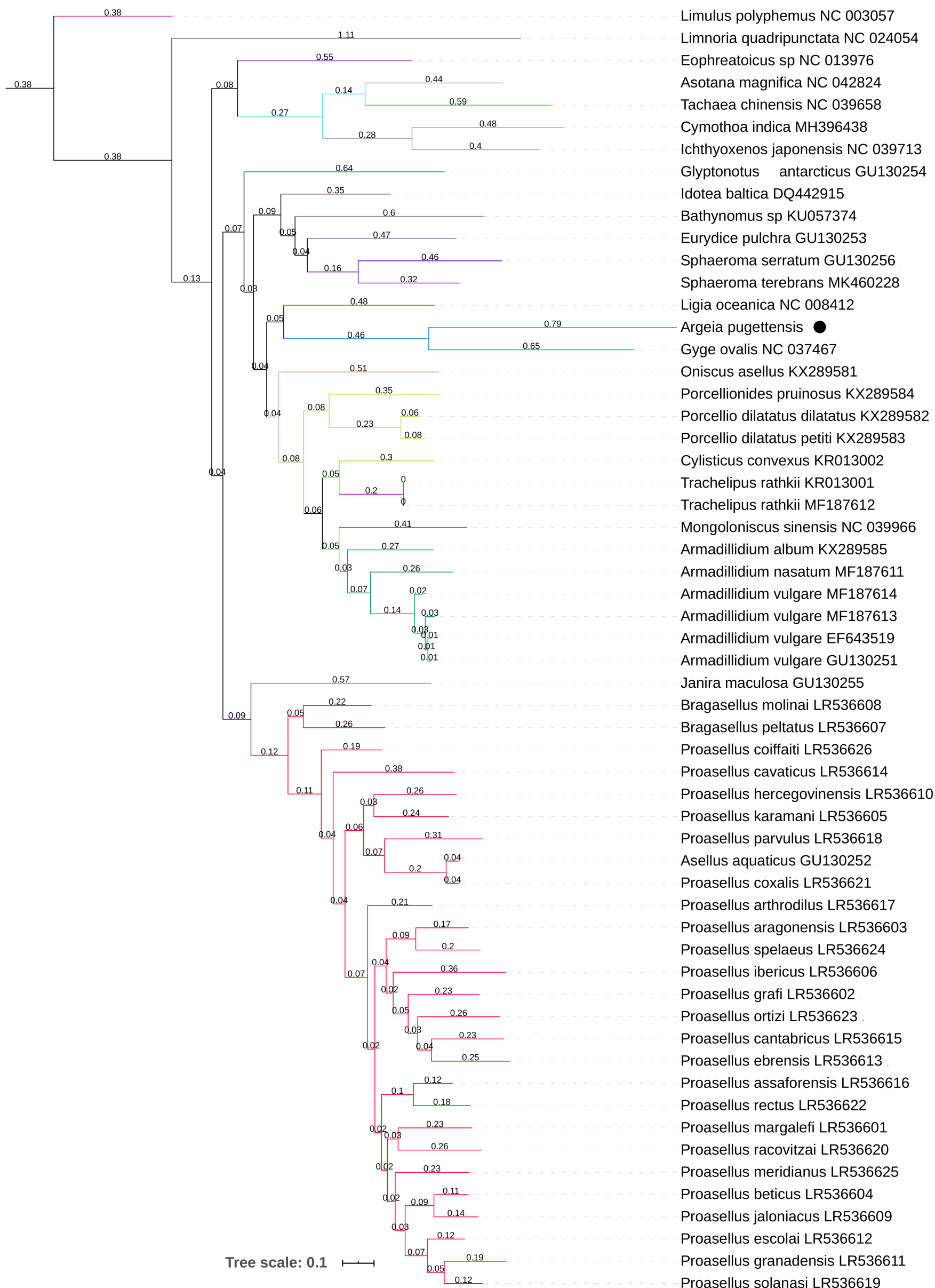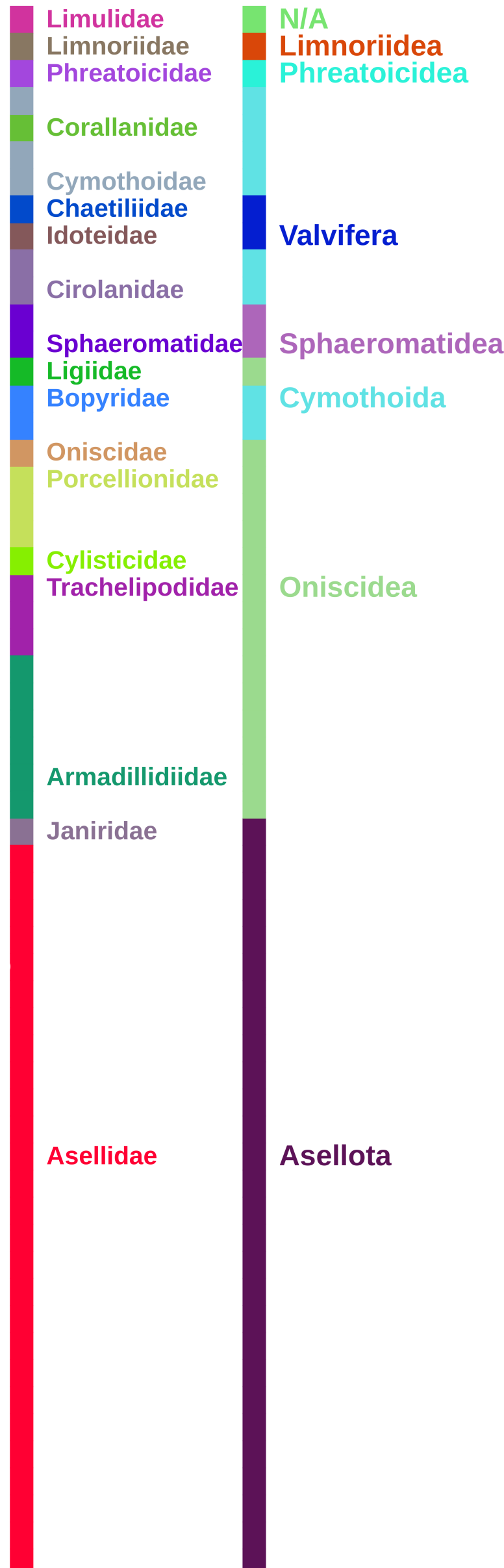

Supplement: Supplementary file 1 — Additional file 1 cox1-based phylogenetic analysis of the entire isopod dataset. The analysis was conducted using Maximum Likelihood algorithm and nucleotide sequences. Lengths are shown on the branches, GenBank numbers next to species names, and taxonomic identity (family and suborder) to the right. The studied specimen is highlighted by a black dot (+ bolded). [file 12864_2020_7021_MOESM1_ESM.pdf]

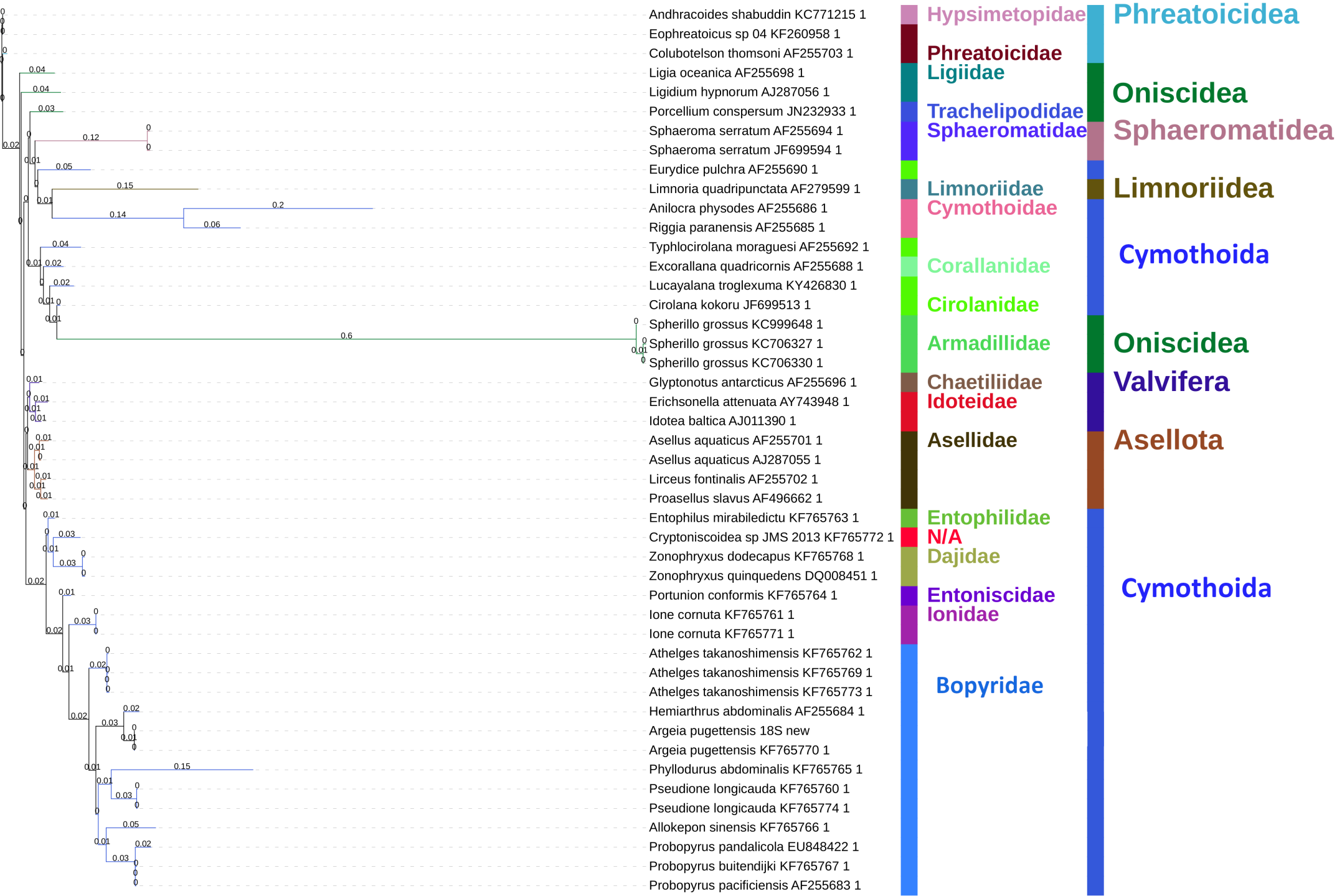

Supplement: Supplementary file 2 — Additional file 2 18S-based phylogenetic analysis of the entire isopod dataset. The analysis was conducted using Maximum Likelihood algorithm and nucleotide sequences. Lengths are shown on the branches, GenBank numbers next to species names, and taxonomic identity (family and suborder) to the right. The studied specimen is named “Argeia pugettensis 18S new”. [file 12864_2020_7021_MOESM2_ESM.pdf]

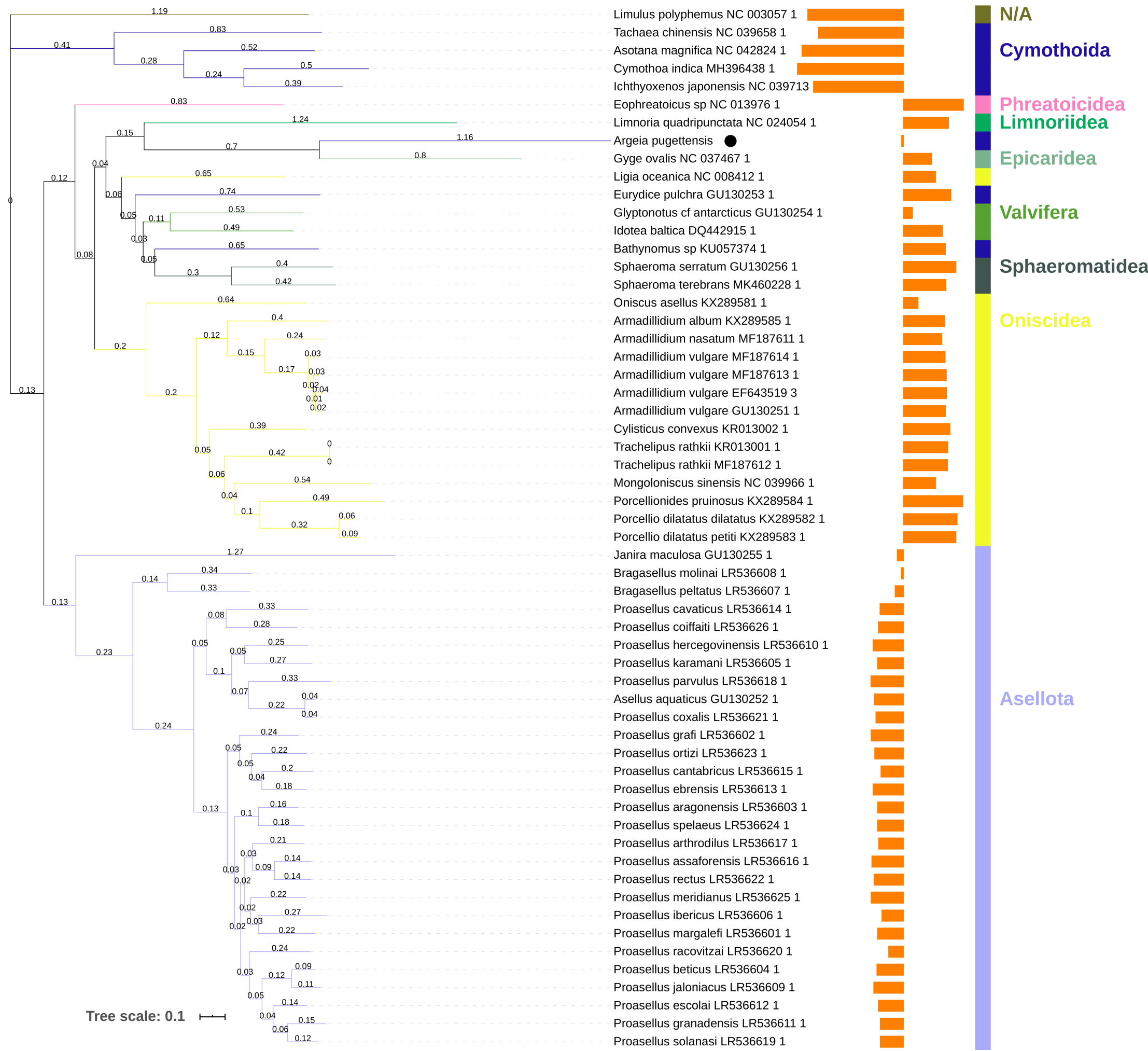

Supplement: Supplementary file 3 — Additional file 3 Branch lengths inferred using mitochondrial phylogenomics of Isopoda (nucleotides dataset). Phylogenetic reconstruction was conducted using nucleotides of concatenated 13 protein-coding genes and ML algorithm. The figure shows (from left to right): a phylogram with branch lengths shown, names of taxa with GenBank numbers for mitogenomes, GC skew on the entire mitochondrial majority strand, and suborder. Limulus polyphemus is the outgroup. [file 12864_2020_7021_MOESM3_ESM.pdf]

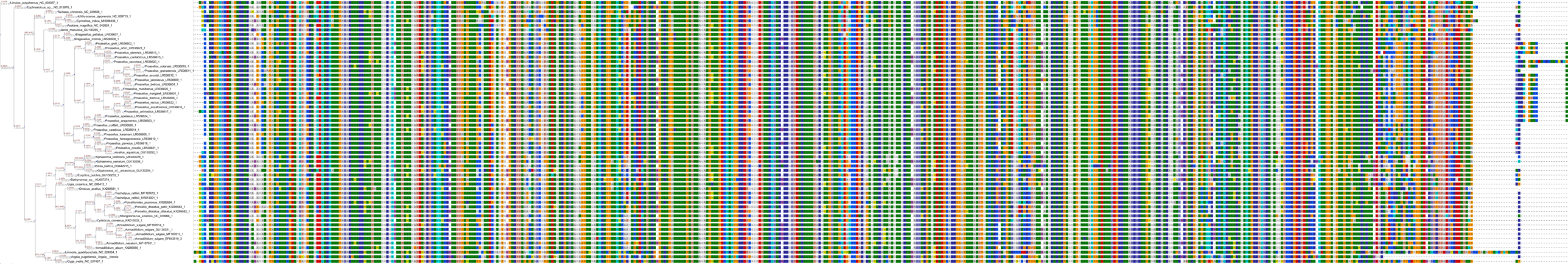

Supplement: Supplementary file 5 — Additional file 5 Nonsynonymous and synonymous mutations in the cox1 gene of isopod mitogenomes. The numbers of nonsynonymous (dN) and synonymous (dS) mutations are shown in grey above the branches (dN/dS), and their ratios (ω) in red, below the branch. The numbers were inferred using free-ratio branch evolutionary model implemented in the ete-evol tool. The phylogram shown in Fig. 3 was used for the analysis. Corresponding GenBank numbers are shown next to species names. [file 12864_2020_7021_MOESM5_ESM.pdf]
