# Supplementary material for: Disrupted architecture and fast evolution of the mitochondrial genome of Argeia pugettensis (Isopoda): implications for speciation and fitness
Source: BMC Genomics. 2020 Sep 3;21:607. doi: 10.1186/s12864-020-07021-y (PMC7469299; doi:10.1186/s12864-020-07021-y)
Supplement: Supplementary file 6 — Additional file 6 Primers, organization, size confirmation, cox1 sequencing chromatogram and palindromic repeat analysis. [file 12864_2020_7021_MOESM6_ESM.docx]

**Supplementary File S1.** Primers, organization, size confirmation, *cox1* sequencing chromatogram and palindromic repeat analysis.

**Table S1. Primers used for amplification and sequencing of the mitochondrial genome and *18S* gene of *Argeia pugettensis.*** Length indicates amplicon length in bases. Primers under ‘nad4L-nad6 fragment’ and ‘walking’ sections were used to corroborate the absence of genes and unique architecture.

| **No.** | **Region** | **Name** | **Sequence (5’-3’)** | **Length** |
| --- | --- | --- | --- | --- |
| **Complete mitogenome** | | | | |
| F1 | *16S* | C-F1 | CTAGAGGATAGAAACCAACC | 485 |
|  |  | C-R1 | CGCAGTATCCTAACTGTGC |  |
| F2 | *16S-COX1* | C-F2 | GTCTTGTCGTCCTTCTAGTG | 3127 |
|  |  | C-R2 | CTATGCGCCATCCTTAGTGC |  |
| F3 | *COX1* | C-F3 | GGATTTGGTAATTGACTTGTC | 698 |
|  |  | C-R3 | GTAGCAGAAGTGAAGTAAGC |  |
| F4 | *COX1-COX2* | C-F4 | GGTTACCTAGGTATAGTTTACG | 1324 |
|  |  | C-R4 | CTTTTCATTGGGATGACAAC |  |
| F5 | *COX2* | C-F5 | CCTGTATACCCTGGATGAAG | 388 |
|  |  | C-R5 | CTCTACGACAATAGGCATG |  |
| F6 | *COX2-12S* | C-F6 | GGTAAAGGCAGATGCTGTAC | 3726 |
|  |  | C-R6 | CCTAGAGTTCTTTCATGTTCG |  |
| F7 | *12S* | C-F7 | GTAGACCTAGGACCAGGCTG | 579 |
|  |  | C-R7 | CTCGTAGTCCGAGAGTGACG |  |
| F8 | *12S-CYTB* | C-F8 | GAAAGTGTACTTGGTAGTAG | 1195 |
|  |  | C-R8 | TCCATCCTTATTTCGTGTTC |  |
| F9 | *CYTB* | C-F9 | GGTAGTACCACTCTGGTTGG | 397 |
|  |  | C-R9 | GCAACTGTTATCACAAACC |  |
| F10 | *CYTB-NAD5-1* | C-F10 | CTCCCCACAACCATTGCACG | 1269 |
|  |  | C-R10 | CGAATTAGCAAGTAAACCCC |  |
| F11 | *NAD5-1* | C-F11 | GTGCCTTTTAGTGCTTGACTC | 533 |
|  |  | C-R11 | CTGTATGGGTTGCTCCACC |  |
| F12 | *NAD5-1-NAD4* | C-F12 | GGGACACTCATGACATCCG | 1319 |
|  |  | C-R12 | CTCACAAGGCTTGACACCTG |  |
| F13 | *NAD4* | C-F13 | TACTCAAGGGCTTCACAG | 332 |
|  |  | C-R13 | CCAGAGCTATGTGGACAAC |  |
| F14 | *NAD4-16S* | C-F14 | CTACGGCCTTATGCAGGTGAC | 3281 |
|  |  | C-R14 | GTTACTTTAGGGATAACAGCG |  |
| ***nad4L-nad6* fragment** | | | | |
| F1 |  | C-NAD4LF1 | CTWABNCCHAGNACHCYRTC | 523 |
|  |  | C-NAD6R1 | GGRTTWGGAVTDARACANRCAC |  |
| F2 |  | C-NAD4LF1 | CTWABNCCHAGNACHCYRTC | 484 |
|  |  | C-NAD6R2 | ATYATHCTBCCYCARAAHA |  |
| F3 |  | C-NAD4LF2 | CCHAGNACHCYRTCWCWNAC | 517 |
|  |  | C-NAD6R1 | GGRTTWGGAVTDARACANRCAC |  |
| F4 |  | C-NAD4LF2 | CCHAGNACHCYRTCWCWNAC | 478 |
|  |  | C-NAD6R2 | ATYATHCTBCCYCARAAHA |  |
| **Walking** | | | | |
| *NAD4L* | | C-NAD4LF1 | CTWABNCCHAGNACHCYRTC |  |
|  |  | C-NAD4LF2 | CCHAGNACHCYRTCWCWNAC |  |
| *NAD6* | | C-NAD6F1 | TDTTYTGRGGVAGDATRAT |  |
|  |  | C-NAD6F2 | GTGYNTGTYTHABTCCWAAYCC |  |
| **18S** |  |  |  |  |
|  |  | F-18S | CTCGTAGTTGGAGTTAGCT | 843 |
|  |  | R-18S | CACTCCTGGTGGTGCCCTTC |  |
| **Size confirmation** | | |  |  |
| F1 | 16S-cytb | C-F2 | GTCTTGTCGTCCTTCTAGTG |  |
|  |  | C-YZR1 | CGTGTTCAAAGATCTGGTAG | 10127 |
|  |  | C-YZR1-0 | CACATCATCTTGCTACACGA | 10213 |
| F2 | cytb-16S | C-F9 | GGTAGTACCACTCTGGTTGG | 6682 |
|  |  | C-YZR2 | CCTAACTGTGCAAAGGTAGC |  |

**Table S2. Organization of the mitochondrial genome of *Argeia pugettensis*.** IGR is intergenic region, where a negative value indicates an overlap, and positive value a non-coding region (NCR). All NCRs > 100 bp are indicated in the Gene column. Duplicated segment is highlighted.

| **Gene** | **Position** |  | **Size** | **IGR** | **Codon** |  |  |
| --- | --- | --- | --- | --- | --- | --- | --- |
|  | **From** | **To** |  |  | **Start** | **Stop** | **Strand** |
| *cox1* | 1 | 1428 | 1428 |  | ATG | TAG | + |
| NCR-1 | 1429 | 1620 | 192 |  |  |  |  |
| *trnL2* | 1621 | 1679 | 59 |  |  |  | + |
| *cox2* | 1680 | 2363 | 684 |  | ATA | TAA | + |
| *trnD* | 2390 | 2449 | 60 | 26 |  |  | + |
| *atp8* | 2450 | 2606 | 157 |  | TTG | T | + |
| *atp6* | 2592 | 3266 | 675 | -15 | ATG | TAA | + |
| *cox3* | 3266 | 3922 | 657 | -1 | ATG | TAG | + |
| NCR-2 | 3923 | 4053 | 131 |  |  |  |  |
| *trnG* | 4054 | 4109 | 56 |  |  |  | + |
| *trnR* | 4109 | 4164 | 56 | -1 |  |  | + |
| *nad3* | 4165 | 4467 | 303 |  | ATT | TAG | + |
| *trnA* | 4511 | 4569 | 59 | 43 |  |  | + |
| *nad1* | 4579 | 5508 | 930 | 9 | ATT | TAG | - |
| *trnL1* | 5509 | 5572 | 64 |  |  |  | - |
| *trnN* | 5569 | 5628 | 60 | -4 |  |  | + |
| *rrnS* | 5629 | 6340 | 712 |  |  |  | + |
| *trnW* | 6341 | 6402 | 62 |  |  |  | + |
| *trnS1* | 6455 | 6518 | 64 | 52 |  |  | - |
| *trnE* | 6515 | 6574 | 60 | -4 |  |  | - |
| NCR-3 | 6575 | 6929 | 355 |  |  |  |  |
| *cytb* | 6930 | 8084 | 1155 |  | ATC | TAG | - |
| *trnT* | 8085 | 8146 | 62 |  |  |  | - |
| *nad5* | 8148 | 9698 | 1551 | 1 | ATA | TAA | + |
| *nad4* | 9692 | 11018 | 1327 | -7 | ATG | T | + |
| *trnH* | 11019 | 11078 | 60 |  |  |  | + |
| *trnF* | 11076 | 11136 | 61 | -3 |  |  | - |
| *nad5-2* | 11161 | 12816 | 1656 | 24 | ATT | TAA | - |
| *trnT-2* | 12878 | 12939 | 62 | 61 |  |  | + |
| *pseudo-cytb* | 12940 | 13151 | 212 |  | ATC | TG | + |
| NCR-4 | 13152 | 13319 | 168 |  |  |  |  |
| *trnS2* | 13320 | 13379 | 60 |  |  |  | + |
| *rrnL* | 13380 | 14550 | 1171 |  |  |  | + |
| *trnV* | 14551 | 14613 | 63 |  |  |  | - |
| *trnQ* | 14623 | 14681 | 59 | 9 |  |  | - |
| *trnM* | 14683 | 14746 | 64 | 1 |  |  | + |
| *nad2* | 14747 | 15733 | 987 |  | ATT | TAA | + |
| NCR-5 | 15734 | 15910 | 177 |  |  |  |  |
| *trnK* | 15911 | 15980 | 70 |  |  |  | - |
| NCR-6 | 15981 | 16311 | 331 |  |  |  |  |
| *trnC* | 16312 | 16360 | 49 |  |  |  | - |
| *trnY* | 16360 | 16421 | 62 | -1 |  |  | - |


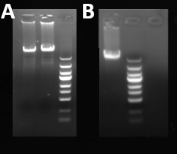


Figure S1. Mitogenomic size confirmation. Additional primers were designed to match cox1 and 16S genes (Table S1 in this file) and amplify the mitogenome in several steps. Panel A: 16S-cytb fragment, where C-F2 to C-YZR1 fragment is in the left lane, and C-F2 to C-YZR1-0 in the right lane. Panel B: cytb-16S fragment (C-F9 to C-YZF2R2). Both panels also exhibit a marker lane (mark: DL5000).


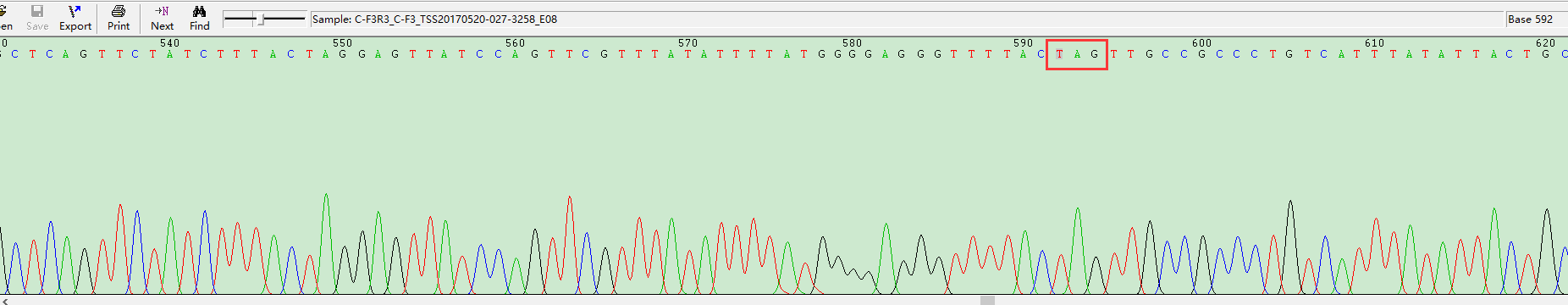


Figure S2. Cox1 sequencing chromatogram. Stop codon causing the truncation of the gene is boxed red.


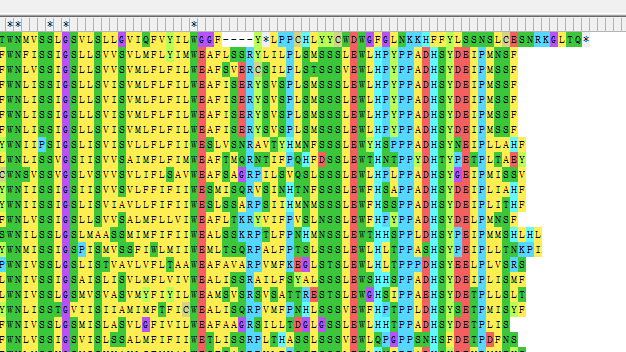


Figure S3. Cox1 alternative stop codon assessment. The topmost sequence belongs to *A. pugettensis*. Stop codons are represented by *. We considered a possibility of TAG stop codon suppression and extension by 40 AAs into the downstream NCR.

Table S3. Palindromic repeat.

| **Position** | **Mismatch** | **Spacer** | **Sequence** |
| --- | --- | --- | --- |
| 8941 | 1 | 13 bp | GTCCTCCTCCGTTCGCCATTTGGACAAAAGA |
|  |  |  | TCTTTTGTCCAAATGGCGAACGGAGGAGAAC |
